# Evaluation of the Antitumour and Antiproliferative Effect of Xanthohumol-Loaded PLGA Nanoparticles on Melanoma

**DOI:** 10.3390/ma14216421

**Published:** 2021-10-26

**Authors:** Magda Fonseca, Ana S. Macedo, Sofia A. Costa Lima, Salette Reis, Raquel Soares, Pedro Fonte

**Affiliations:** 1 Department of Biomedicine, Faculty of Medicine, University of Porto, Al Prof Hernani Monteiro, 4200-319 Porto, Portugal; magdapcfonseca@zonmail.pt (M.F.); raqsoa@med.up.pt (R.S.); 2 i3S, Instituto de Investigação e Inovação em Saúde, Universidade do Porto, Rua Alfredo Allen, 208, 4200-135 Porto, Portugal; 3 LAQV, REQUIMTE, Department of Chemical Sciences-Applied Chemistry Lab, Faculty of Pharmacy, University of Porto, Rua de Jorge Viterbo Ferreira 228, 4050-313 Porto, Portugal; anatmacedo@hotmail.com (A.S.M.); aclima.sofia@gmail.com (S.A.C.L.); shreis@ff.up.pt (S.R.); 4 Center for Marine Sciences (CCMAR), University of Algarve, Gambelas Campus, 8005-139 Faro, Portugal; 5 Department of Chemistry and Pharmacy, Faculty of Sciences and Technology, University of Algarve, Gambelas Campus, 8005-139 Faro, Portugal; 6 iBB—Institute for Bioengineering and Biosciences, Department of Bioengineering, Instituto Superior Técnico, Universidade de Lisboa, 1049-001 Lisboa, Portugal; 7 Associate Laboratory i4HB—Institute for Health and Bioeconomy at Instituto Superior Técnico, Universidade de Lisboa, Av. Rovisco Pais, 1049-001 Lisboa, Portugal

**Keywords:** melanoma, xanthohumol, PLGA nanoparticle, macrophage, antitumour, antiproliferative, drug delivery, cancer

## Abstract

Cutaneous melanoma is the deadliest type of skin cancer and current treatment is still inadequate, with low patient survival rates. The polyphenol xanthohumol has been shown to inhibit tumourigenesis and metastasization, however its physicochemical properties restrict its application. In this work, we developed PLGA nanoparticles encapsulating xanthohumol and tested its antiproliferative, antitumour, and migration effect on B16F10, malignant cutaneous melanoma, and RAW 264.7, macrophagic, mouse cell lines. PLGA nanoparticles had a size of 312 ± 41 nm and a PdI of 0.259, while achieving a xanthohumol loading of about 90%. The viability study showed similar cytoxicity between the xanthohumol and xanthohumol-loaded PLGA nanoparticles at 48 h with the IC50 established at 10 µM. Similar antimigration effects were observed for free and the encapsulated xanthohumol. It was also observed that the M1 antitumor phenotype was stimulated on macrophages. The ultimate anti-melanoma effect emerges from an association between the viability, migration and macrophagic phenotype modulation. These results display the remarkable antitumour effect of the xanthohumol-loaded PLGA nanoparticles and are the first advance towards the application of a nanoformulation to deliver xanthohumol to reduce adverse effects by currently employed chemotherapeutics.

## 1. Introduction

Skin cancer is the most prevalent type of malignancy in the Caucasian population of developed countries, and cutaneous melanoma is responsible for, approximately 80% of skin cancer related deaths [1,2]. The incidence of cutaneous melanoma has grown annually worldwide, with an increase rate of about 0.6% [3,4]. Melanoma is caused by the uncontrolled division of melanocytes, [5,6], and it recruits immune cells from the external environment, to integrate into the tumour microenvironment and to provide immunoprotection and faster progression and metastasis [7,8,9,10]. The macrophages are one of the most abundant immune cell type found in the tumour microenvironment [11,12,13], the tumour associated macrophages (TAM), acquiring a predominant M2 phenotype (alternative macrophage activation) or pro-tumoural phenotype [14]. In a smaller proportion, the M1 phenotype (classical macrophage activation) is present and it exerts antitumour action [15]. This phenomenon is called macrophage reverse polarization [16].

An early-stage localized cutaneous melanoma can be successfully removed by surgical treatment with a high survival rate (97–99.8%) [17], but if not removed, it can metastasize and other conventional therapies, such as chemotherapy, radiation therapy, targeted therapy and/or immunotherapy, must be administered [18]. The personalized chemotherapies are inefficacious, because the most commonly used drugs, such as dabrafenib, trametinib, and encorafenib [19] have been found to extend remission-free period of time by only a few months, leading to the development of drug resistance and death [20,21,22,23]. Innovative strategies for treatment of cutaneous melanoma have been investigated and developed in the last decades. Phytochemicals with biological activity have become considered advantageous alternatives [24,25], mainly due to their higher safety and minimal toxicity, as is the case of polyphenols [26,27,28,29], which improve intracellular redox balance and exert a preventive activity on oxidative stress [30,31].

Xanthohumol (XN) from the hop plant (*Humulus lupulus* L.) has demonstrated a good bioactivity on the prevention and treatment of cutaneous melanoma, acting by inhibiting both tumourigenesis and metastasis [32]. Moreover, XN may work synergistically with the current conventional treatments to decrease the doses that often result in toxicity and severe side effects. However, XN presents poor water solubility and bioavailability, low stability, high photosensitivity and a short half-life [33,34]. Thus, the encapsulation of XN into nanocarriers is potentially an efficient strategy to overcome these limitations [35].

Polymeric nanoparticles are good carriers to load polyphenols due to their versatility, biocompatibility and biodegradability properties [36,37,38]. One of the most used polymers to produce nanoparticles is poly-lactic-co-glycolic acid (PLGA), and it has already been approved for the use in humans by the Food and Drug Administration (FDA) [39]. PLGA nanoparticles are excellent carriers to encapsulate drugs and deliver them in a sustained, controlled or even targeted manner [40,41,42,43,44]. An increasing interest in the design of polyphenols-loaded PLGA nanoparticles to be applied on tumour imaging [45] and targeted delivery [46,47,48,49,50] has been noticed in the literature. Previously, polyphenol-loaded PLGA nanoparticles showed a biphasic release, with an initial burst release to 16 h, followed by a controlled release of up to three days [51]. This demonstrates the potential of PLGA nanocarriers to deliver polyphenols in a sustained manner, decreasing the number of administrations needed, which ultimately may improve patient treatment compliance.

The main aim of this work is to evaluate the antitumour and antiproliferative effect of XN loaded into PLGA nanoparticles in cutaneous melanoma. Therefore, a new approach in melanoma treatment is proposed. Herein, the XN will be encapsulated into nanocarriers that will be characterized and tested for efficacy in preventing the progression and metastization of tumoural melanocytes.

## 2. Materials and Methods

### 2.1. Materials

PLGA 50:50 (Purasorb^®^ PDLG 5002A) was a gift from Corbion Purac (Amsterdam, The Netherlands) and was used to produce nanoparticles. Dichloromethane and polyvinyl alcohol (PVA, MW 89,000–98,000 Da) were from Sigma-Aldrich (St. Louis, MO, USA). The synthetic XN was from HWI Pharma Services (Rülzheim, Germany). The milli-Q water was prepared in-house. For cell culture studies, B16F10 (ATCC CRL-6475) and RAW 264.7 (ATCC TIB-71) (ATCC, Manassas, VA, USA) mouse cell lines were used. Dulbecco’s modified Eagle’s medium (DMEM), heat-inactivated fetal bovine serum (FBS), penicillin/streptomycin/amphotericin B were from Sigma-Aldrich (St Louis, MO, USA) and used for cell culture media. The CD86 monoclonal rabbit anti-mouse from ABclonal (Massachussets, MA, USA), and the CD206 monoclonal rat anti-mouse from ThermoFisher Scientific (Waltham, MA, USA) were also used. Alexa Fluor 594 conjugated goat anti-rabbit antibody and the Alexa Fluor 555 conjugated donkey anti-rat antibody were purchased from Abcam, UK.

### 2.2. Production of XN-Loaded PLGA Nanoparticles

The XN-loaded PLGA nanoparticles were produced using an adapted oil in water (O/W) emulsion technique described by Fonte et al. [43,44]. Briefly, 2 mg of XN were added to 8 mg of PLGA (50:50) and dissolved in 1 mL of dichloromethane, and then poured to 2 mL of PVA 2% (*w*/*v*). This mixture was sonicated using a Q125 Sonicator from QSonica Sonicators (Newtown, CT, USA) for 30 s at 70% of amplitude. Then, the dichloromethane was removed by evaporation at magnetic stirring during 3 h. Both XN-loaded PLGA nanoparticles and unloaded PLGA nanoparticles were produced in triplicate.

### 2.3. Particle Size, Polydispersity Index and Zeta Potential Characterization of XN-Loaded PLGA Nanoparticles

The particle size and polydispersity index (PdI) were analyzed by dynamic light scattering using a Malvern® Zetasizer Nano ZS (Worcestershire, UK) after proper dilution with milli-Q water. The zeta potential was evaluated by the electrophoretic mobility analysis using the same equipment. All samples were analysed in triplicate at 23 ± 2 °C.

### 2.4. Association Efficiency (AE) and Loading Capacity (LC) of XN

The XN-loaded PLGA nanoparticles were centrifuged at 20,000 rpm for 20 min using a Centrifuge 5810 R from Eppendorf International (Hamburg, Germany), and the supernatant was collected. The XN present in the supernatant was quantified by UV spectroscopy using a UV–VIS Spectrophotometer Evolution^®^ 300 from Thermo Scientific (Hertfordshire, England) at its maximum absorption wavelength (370 nm). The AE and LC were calculated using the following Equations:

(1) $$\mathrm{AE}\;=\;\frac{\mathrm{Total amount of xanthohumol}\;-\;\mathrm{Free amount of xanthohumol in the supernatant}}{\mathrm{Total amount of xanthohumol}}$$

(2) $$\mathrm{LC}\;=\;\frac{\mathrm{Total amount of xanthohumol}\;-\;\mathrm{Free amount of xanthohumol in supernatant}}{\mathrm{Total weight of nanoparticles}}$$

### 2.5. Attenuated Total Reflectance Fourier Transform Infrared Spectroscopy (ATR-FTIR)

The obtained XN-loaded PLGA nanoparticles were freeze-dried and evaluated by ATR-FTIR in a PerkinElmer^®^ Spectrum 400 (Waltham, MA, USA) equipped with an attenuated total reflectance (ATR) device. The spectra were obtained by collecting 100 scans of each sample, between 4000 and 600 cm^−1^, with a resolution of 4 cm^−1^. The FTIR analysis was also performed for XN and other control samples.

### 2.6. Scanning Electron Microscopy (SEM)

The samples were previously washed with milli-Q water using a Hermle Z 32 HK centrifuge from Hermle LaborTechnik (Wehingen, Germany) at 12,600× *g* for 15 min at 4 °C. The formulations were placed on metal stubs and vacuum-coated with a layer of gold/palladium during 20 s with a current of 25 mA. The SEM analysis was performed on a JSM-7001F from JEOL (Tokyo, Japan).

### 2.7. Cell Culture

B16F10 (ATCC CRL-6475) and RAW 264.7 (ATCC TIB-71) cell lines from mice were used. The B16F10 cell line was originally isolated from the cutaneous melanoma lung metastases of C57Bl6/J mice, and consists of high tropism metastatic cutaneous melanoma cells for lung invasion. The RAW 264.7 cell line consists of undifferentiated macrophages isolated from the tumour induced in Balb/c mice by the Abelson leukemic virus. Both cell lines were grown in DMEM supplemented with 10% (*v*/*v*) FBS and 1% (*v*/*v*) penicillin/streptomycin/amphotericin B. The cultures were kept in a 37 °C incubator in a humidified atmosphere with 5% CO_2_. An extra 10% (*v*/*v*) heat-inactivated FBS volume was added to each standard 5 mL plate to the RAW 264.7 culture to optimize cell growth. Cellular manipulation was performed until reaching 70 to 80% confluence.

### 2.8. Cell Culture Studies

B16F10 cells were plated in 96- and 24-well plates, with an amount of 1 × 10^4^ cells per well. RAW 264.7 cells were plated in 24-well plates, with an amount of 2 × 10^4^ cells per well. In the case of RAW 264.7 cells, an extra volume of 10% FBS was added to each well to increase cell adhesion. The cell adhesion occurred during 24 h for both cell lines. XN was dissolved in ethanol and then added to cell culture medium at various concentrations (2, 4, 6, 8, 10, 20, 30, and 40 µM), XN 14 µM solution, XN-loaded PLGA nanoparticles suspension, and PLGA nanoparticles suspension with a concentration equivalent to 14 µM. Ethanol concentrations in cell culture were kept below 0.1% in every culture. All the treatments used DMEM without supplementation of FBS. The incubation occurred between 24 h and 93 h. In the case of the RAW 264.7 incubation protocol with the 3 types of treatments described, the respective conditioned media (CM) were collected after 48 h of incubation and kept at −20 °C for analysis.

### 2.9. Cell Viability Assay

The 3-(4,5-dimethylthiazol-2-yl)-2,5-diphenyltetrazolium bromide (MTT) was added to each well, 20 and 80 µL for 96- and 24-well plates respectively, after the incubation of B16F10 cells with the treatments described in Section 2.8. The control used was the same amount of cells per well incubated in DMEM only. The incubation of the plates occurred at 37 °C for 3 h. The absorbance was measured at 550 nm and 650 nm, and the latter wavelength corresponding to the blank assay, on ThermoElectron Corporation, Multiskan Ascent (Berverly, MA, USA) plate reader. The cell viability was determined based on the difference between the absorbances obtained at the two wavelengths used and on their variation proportional to the MTT concentration in each well, normalizing the percentage of viability to the control.

### 2.10. Wound Healing Experiment

In each well, a vertical slit with the micropipette tip was made after 24 h of B16F10 cells adhesion and 95% confluence, and immediately before the treatments were applied. The control used was the same amount of cells per well incubated in DMEM only. Photographs were taken of each well using an inverted Nikon Eclipse 50i microscope Nikon Europe B.V., Amsterdam, The Netherlands) with a magnitude of 200×. After 48 h of incubation with the different solutions and suspensions, new photographs were taken of each well under similar conditions. The calculation of the percentage of migration of B16F10 cells was performed in the Image J software.

### 2.11. Fluorescence Immunocytochemistry for Detection of Membrane Markers

An immunocytochemistry assay was performed for each marker (CD86 and CD206). RAW264.7 cells were fixed with 4% paraformaldehyde solution in phosphate buffer saline (PBS) for 15 min at room temperature, followed by a series of 3 washes with PBS. Nonspecific binding was blocked with a blocking solution consisting of 10% FBS, 1% bovine serum albumin (BSA) and 0.3 M glycine in PBS for 1 h at room temperature and in a humidified chamber. The incubation with the CD86 monoclonal rabbit anti-mouse primary antibody A10795 and the CD206 monoclonal rat anti-mouse primary antibody MA5-16871 at 1:50 and 1:100 dilution, respectively, was performed in blocking serum, overnight at 4 °C in a humidified chamber, maintaining the negative controls with the blocking solution. Then, the incubation with secondary antibodies, namely Alexa Fluor 594 conjugated goat anti-rabbit antibody (Abcam, Cambridge, UK, ab150084) and Alexa Fluor 555 conjugated donkey anti-rat antibody ab150150, at a dilution of 1:150 and 1:200, respectively, in blocking serum, was carried out for 2 h at room temperature in a humidified chamber. Cell nuclei were stained with 1:100 DAPI for 15 min at room temperature and in a humidified chamber. The mounting was made with a solution of 1:3 PBS/glycerol and the cells were observed under the “ZEISS” fluorescence microscope with the “Apotome” system and the “AxioVision” program (Carl Zeiss MicroImaging GmbH, Copenhagen, Denmark), with a magnitude of 200×. Image J software was used to quantify red fluorescence for each membrane marker.

### 2.12. Statistical Analysis

The statistical analysis of the results was performed using the IBM SPSS Statistics software version 26 (IBM, Armonk, NY, USA). The non-parametric test Kruskal-Wallis for independent samples was used, considering *p* = 0.050 as the level of statistical significance. Statistically significant differences are given by *p* values < 0.050. All quantitative values are expressed as mean ± standard error of the mean (S.E.M.) for cell culture studies, and mean ± standard deviation (SD) for nanoparticle characterization studies of three replicates.

## 3. Results and Discussion

### 3.1. Particle Size, Polydispersity Index, Zeta Potential, AE and LC

The particles had a size between 250–320 nm, a PdI below 0.3, and a zeta potential of approximately −20 mV (Table 1). The production method showed the formulations had great reproducibility. These results are in accordance with results previously reported, where the small size, unimodal distribution and negative surface charge result in greater particle-particle repulsion, thereby increasing the formulation stability [43]. It was observed that the PLGA nanoparticles both with and without loaded XN had similar size and PdI, demonstrating the robustness of the production method [52]. A more negative zeta potential for XN-PLGA nanoparticles was also observed, mostly due to the drug being adsorbed onto the nanoparticles surface.

The XN AE of the formulation was about 90% (Table I), which is a remarkable achievement when compared to recent literature reporting a AE of about 13% for XN loaded into PLGA nanoparticles [53]. Again, this shows the robustness of the nanoparticle production method. Approximately 16% of the LC was higher than the LC values reported in other studies using flavonoids encapsulated in PLGA nanoparticles, showing the feasibility of this technique and PLGA nanoparticles for the encapsulation of XN [44]. Using the developed method, hydrophobic drugs, such as XN, do not suffer relevant leakage from the polymeric matrix to leading to high XN content in the nanoparticles, which is very important from the biological and industrial points of view [54].

### 3.2. Interaction of XN and PLGA Nanoparticles Assessed by ATR-FTIR

**Figure 1:**
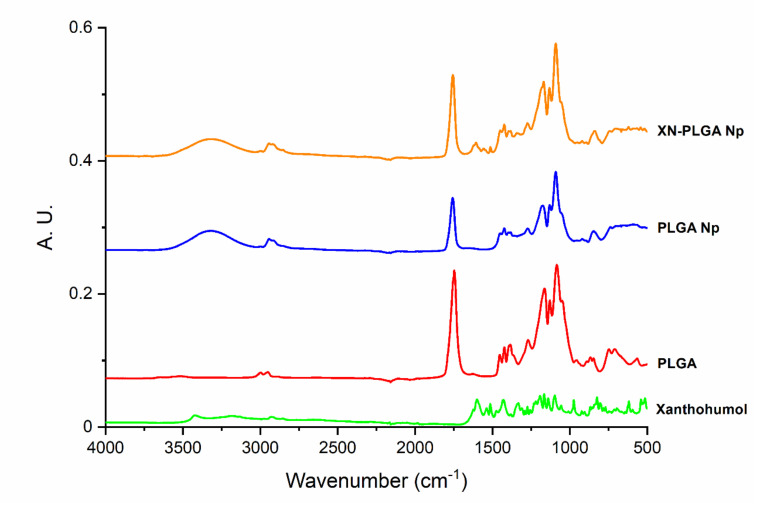
ATR-FTIR spectra of XN, PLGA, PLGA nanoparticles (PLGA Np), and XN-loaded PLGA nanoparticles (XN-PLGA Np).

The ATR-FTIR technique was used to understand the nature of the interactions of XN and the PLGA nanoparticles. The analysis of FTIR spectra was collected for XN and PLGA to serve as controls, the PLGA nanoparticles, and the XN-loaded PLGA nanoparticles (Figure 1) after the formulations were freeze-dried. A typical peak for the PLGA between 1750–1760 cm^−1^ was found, characteristic of the C=O stretching at 3000 cm^−1^, corresponding to the C–H stretching [43]. The pure XN shows the -OH vibration band of the chalcone groups at 3400 cm^−1^, which disappears or can no longer be seen due to the band present in the PLGA nanoparticles spectrum at the same wavelength. The C=O stretching spectral band at 1626 cm^−1^, and –C=C– vibrations at 1545 cm^−1^ and 1514 cm^−1^ due to phenol and hydroxyl-benzoyl fractions were also observed in the XN-loaded PLGA nanoparticles [44]. This observation points out to the existence of an interaction between XN and polymeric nanoparticles, showing that XN remains associated with PLGA nanoparticles and thus efficiently loaded.

### 3.3. SEM Analysis

**Figure 2:**
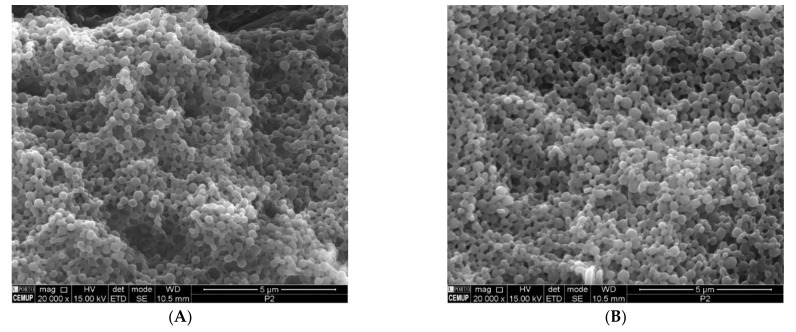
SEM microphotographs of unloaded nanoparticles (**A**) and XN-loaded nanoparticles (**B**) after freeze-drying (at 20,000×).

The morphology of the nanoparticles was observed by SEM to evaluate the size, shape, and surface of the nanoparticles. This information is relevant to predict the stability of nanoparticles and, consequently, of the encapsulated XN. The nanoparticles were purified prior to the SEM analysis to remove the PVA, as it hides the nanoparticles upon gold/palladium coating, hindering the observation of the nanoparticle morphology. The SEM images showed that the nanoparticles had a spherical shape and smooth surface, both for the unloaded and XN-PLGA nanoparticles, showing that encapsulating XN does not affect the size, shape, and surface of the nanoparticles (Figure 2). The spherical shape and smooth surface is also characteristic of the PLGA nanoparticle matrix and encapsulating method used [43,44]. In addition, the particle size observed in the images is in accordance with the results obtained for the particle size measurements.

### 3.4. B16F10 Viability Study with XN Solutions

**Figure 3:**
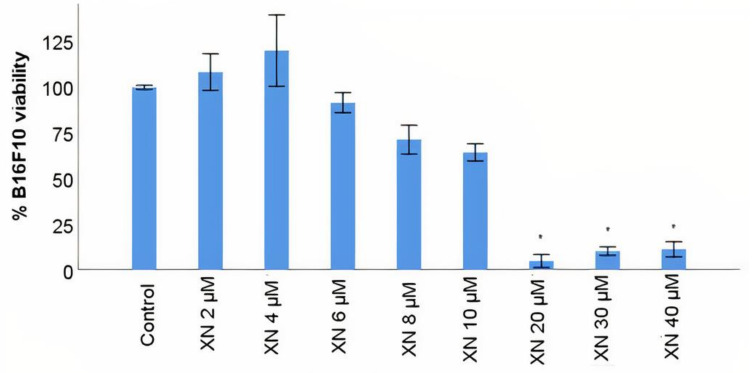
B16F10 viability study with solubilized XN in different concentrations. The control refers to B16F10 incubated with DMEM. “XN” stands for XN. B16F10 viability is expressed in mean percentage ± S.E.M. (*n* = 3 for each treatment assay). * Statistically different at a level of *p* < 0.050 in comparison with the control.

The use of XN as a potential cytotoxic phytochemical for cancer cells has been studied in recent years [55,56] and, in particular, for melanoma tumours [57,58]. The cytotoxicity of XN was evaluated in the B16F10 malignant cutaneous melanoma cell line using the MTT assay, with a range of concentrations between 2 and 40 µM based on some previous research done by other groups [57,58]. The viability of B16F10 cells relative to control (B16F10 cells incubated with DMEM) was gradually reduced from 4 µM to 10 µM concentration. At the concentration of 20 µM, XN induced a sharp decrease of about 70% in the viability of the tumour melanocytes, maintaining the viability in the order of 10% with the remaining concentrations tested (Figure 3), with statistically significant differences relative to control (*p* < 0.05). The results suggested that XN is cytotoxic to the malignant cutaneous melanoma cell line B16F10 in a dose-response manner, achieving just 10% of cell viability at 20 µM. Interestingly, with twice of the concentration of 20 µM there is not a decrease in viability, making 20 µM a concentration enough and better to decrease potential side effects to healthy cells when compared with 40 µM. The IC50 for XN is, approximately, 10 µM according to AAT Bioquest software for IC50 calculation.

### 3.5. B16F10 Viability Study with XN-Loaded PLGA Nanoparticles Compared to XN Solubilized Form

**Figure 4:**
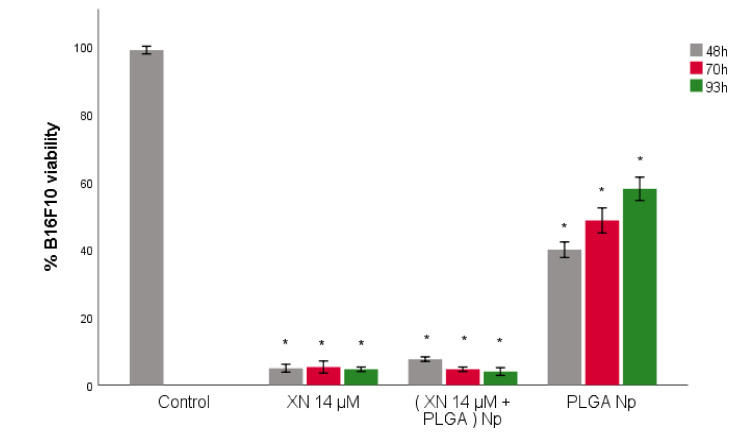
B16F10 viability assay with XN-loaded PLGA nanoparticles compared to XN solubilized form. The control refers to B16F10 incubated with DMEM. “XN”, “Np” and “PLGA” stands for XN, nanoparticles and poly-lactic-co-glycolic acid, respectively. B16F10 viability is expressed in mean percentage ± S.E.M. (*n* = 3 for each treatment assay). * Statistically different at a level of *p* < 0.050 in comparison with the control.

XN has physicochemical characteristics that limit its administration in the solubilized and free form [33]. The encapsulation of XN in nanotechnological vehicles has been tested [32], suggesting a very promising alternative for the controlled, prolonged and specific distribution of XN to the tumour site. PLGA is the only FDA-approved biomaterial for the production of therapeutic nanosystems [39], but the scientific literature reveals very few studies testing the antitumour efficacy of XN delivered by polymeric PLGA nanoparticles. Using the same in vitro model of malignant cutaneous melanoma, the cytotoxic action of XN was evaluated in B16F10 tumour melanocytes when encapsulated into PLGA polymeric nanoparticles and compared to that of XN in solution (non-encapsulated) by measuring the viability of B16F10 using MTT assay. The XN concentration of 14 µM was used instead of 20 µM (maximum cytotoxic effect, according to Figure 3), because it was closer to the IC50 (10 µM). A slightly higher concentration than the IC50 was used. On one hand, a sharp reduction of B16F10 viability (between 10 and 20 µM) was desired. On the other hand, there are some studies where concentrations of XN up to 15 µM with maximum cytotoxic effect were tested [55]. Thus, the 14 µM value was defined as the XN concentration to be used in the following assays. According to Figure 4, the viability of B16F10 tumour melanocytes was measured in three time periods of incubation of cells with the treatments (XN solution 14 µM–XN 14 µM, PLGA-loaded nanoparticles with XN 14 µM–(XN 14 µM + PLGA) Np–and PLGA nanoparticles–PLGA Np): at 48, 70 and 93 h. The 93 h time period was used to verify the previous result. Two controls were used, one with untreated B16F10 and one with B16F10 treated with PLGA nanoparticles. PLGA nanoparticles released encapsulated XN between 48 h and 93 h of incubation of B16F10 tumour melanocytes. The cytotoxicity of XN to B16F10 was statistically identical to that of non-encapsulated XN in the same molar concentration and during the same time period (Figure 4), with statistically significant differences relative to the control (B16F10 incubated with DMEM). Regarding the temporal study, the 48 h time-point seems to be the most appropriate for the subsequent assays, although there are no statistically significant differences between the three time periods tested. This shorter time-point of 48 h ensured that the encapsulated XN is released from the nanosystem, with the advantage that false viability reduction—the reduction on B16F10 viability due to natural death in conditions of prolonged culture—is not considered in these results. We also observed that there are components of PLGA nanoparticles that can stimulate the increase on the viability of B16F10, with statistically significant differences relative to control (B16F10 incubated with DMEM) (Figure 4). This assay was intended to be a negative control from which the observed variation in the viability of B16F10 was not expected. The components of PLGA nanoparticles that may contribute to the increased viability observed may be the PVA and/or the PLGA polymer itself. PLGA should be pharmacologically inert according to its FDA approval for nanotechnology application. The result suggests that B16F10 cells may metabolize the biodegradable and biocompatible polymer using the resulting products to grow. This could be a mechanism of metabolic adaptation of B16F10 and, consequently, become a possible strategy of resistance to the cytotoxic effect caused by XN-loaded PLGA nanoparticles. In fact, cutaneous melanoma is a tumour that easily develops resistance to targeted therapies through mechanisms not yet fully understood. One possible test to evaluate the effect of PVA on B16F10 viability is to produce PLGA nanoparticles with another surfactant whose effect on tumour melanocyte viability is known and to compare B16F10 responses to the different surfactants. The results suggested that PLGA nanoparticles released encapsulated XN and the cytotoxic action of this polyphenol to B16F10 is statistically similar to that of XN in its non-encapsulated form, and that the most appropriate time period for controlled release of XN from PLGA nanoparticles is 48 h. To quantify the amount of XN released from PLGA nanoparticles, release studies must be performed.

### 3.6. B16F10 Migration Study with XN-Loaded PLGA Nanoparticles Compared to XN Solubilized Form

**Figure 5:**
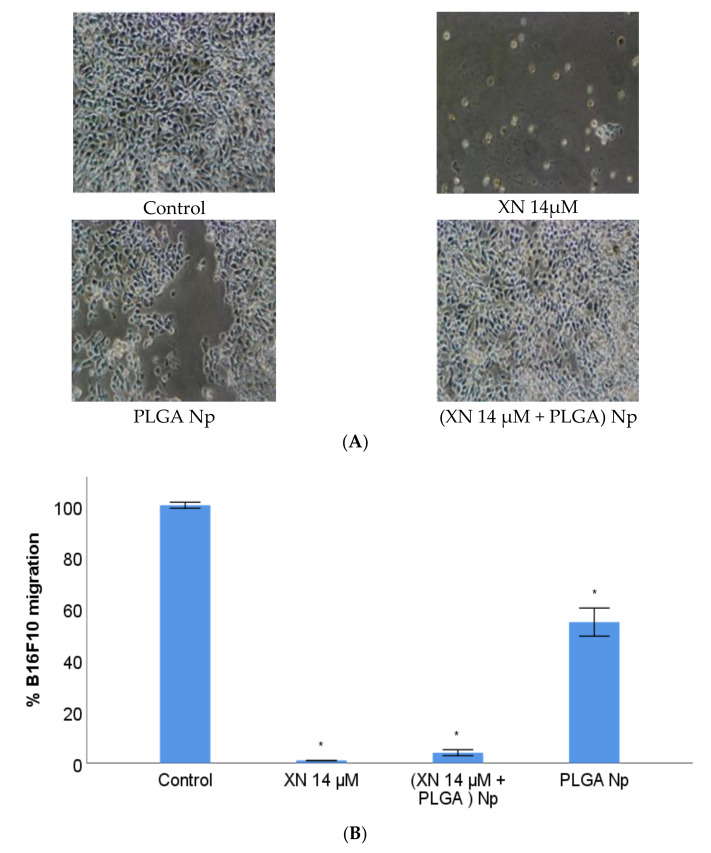
B16F10 Wound Healing assay with XN-loaded PLGA nanoparticles compared to XN solubilized form after 24 h treatment. The control refers to injury assay of B16F10 cells incubated with DMEM. “XN”, “Np”, and “PLGA” stands for XN, nanoparticles and poly-lactic-co-glycolic acid, respectively. (**A**) Representative inverted optical microscopic images, 200×. (**B**) Bars represent B16F10 cells migration towards the injured space. B16F10 migration is expressed in mean percentage ± S.E.M.(*n* = 3 for each treatment assay). * Statistically different at a level of *p* < 0.050 in comparison with the control.

**Figure 6:**
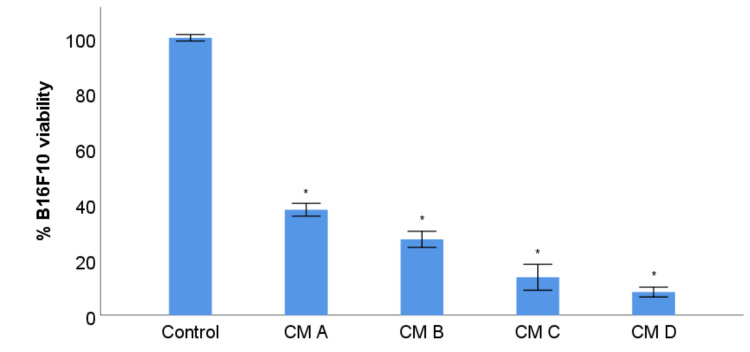
B16F10 viability study with CM from stimulation of RAW 264.7 with XN-loaded PLGA nanoparticles and XN solubilized form. The control refers to B16F10 incubated with DMEM. B16F10 cells treated with: CM from RAW 264.7 incubated with DMEM (A), CM from RAW 264.7 incubated with XN solution 14 µM (B), CM from RAW 264.7 incubated with XN-loaded PLGA nanoparticles (C), CM from RAW 264.7 incubated with PLGA nanoparticles (D). B16F10 viability is expressed in mean percentage ± S.E.M.(*n* = 3 for each treatment assay). The experiment was repeated three times. * *p* < 0.050 in comparison with the control.

The ability of XN to inhibit the migration of B16F10 tumour melanocytes, in the free and in the nanoencapsulated form, at a concentration of 14 µM and at the time period of 48 h of incubation of B16F10 with the treatments, was evaluated by the “Wound Healing” assay (Figure 5). Previous in vitro assays to evaluate the ability of XN to inhibit migration of cells from different tumour lines have revealed that this polyphenol may be used on the prevention of metastisation, because it suppresses the migratory phenotype of diverse tumour cell types [59,60]. Based on these results, the next step was to understand whether the same effect occurs with the malignant cutaneous melanoma cell line B16F10, as reported. Since the evaluation of the therapeutic and preventive potential of an XN nanosystem is the main goal of this research, this assay also tested nanoencapsulated XN. The concentration value and incubation time used were obtained from the assays previously performed. XN inhibited the migration of B16F10 tumour melanocytes, either in the non-encapsulated and in the encapsulated form by PLGA nanoparticles (Figure 5), with no statistically significant differences between these two XN formulations (Figure 5B). Regarding the control (B16F10 incubated with DMEM), there are statistically significant differences in the inhibitory activity of these two forms of XN, as shown in Figure 6. We also observed that there are components of PLGA nanoparticles non-loaded with XN that can stimulate an increased migration of B16F10 (Figure 5B). This result was similar to that obtained from the viability assay and it was also unexpected. The same discussion can be approached, as well as the future research perspectives about studying the stimulatory effect on migration caused by PLGA and/or PVA. The results suggest that PLGA nanoparticles are efficient vehicles of XN, favoring their inhibitory action on the migration of B16F10 tumour melanocytes, in a statistically identical way to XN in its free form.

### 3.7. B16F10 Viability Study with Conditioned Media from Stimulation of RAW 264.7 with XN-Loaded PLGA Nanoparticles and XN Solubilized Form

The tumour microenvironment plays a crucial role in cancer progression. Understanding the interactions between tumour cells and the surrounding microenvironmental cells (immune, endothelial, epithelial, etc), as well as the interactions with nonliving elements such as the extracellular matrix [61,62] allows us to define new therapeutic and cancer prevention strategies [63]. Thus, it was essential to investigate the biological action of XN on macrophages, a type of immune cells very often present in the microenvironment of any tumour [64], along with studying the direct effect of XN on viability and migratory capacity of tumour melanocytes. Previous studies suggested that XN has a diverse biological action on macrophages under pathological conditions other than cancer [65,66]. Few reports have focused on the interaction between XN and macrophages in cancer [67]. Therefore, the investigation performed on malignant cutaneous melanoma becomes a pertinent innovation. An inherent biological function of macrophages is their ability to change their phenotype polarization depending on whether external stimuli are more or less inflammatory, acquiring specific membrane receptors characteristic of an anti-tumour (M1) or pro-tumour (M2) phenotype, as noted above. Based on this evidence, the next step was to evaluate the immunomodulatory activity of XN in the non-encapsulated and in the nano-encapsulated form, on the RAW 264.7 macrophage cell line, by quantifying the viability of the B16F10 tumour melanocytes using the MTT assay. B16F10 cells were treated with CM from RAW 264.7 cultures incubated with XN solution 14 µM and suspensions of XN-loaded PLGA nanoparticles and non-loaded PLGA nanoparticles.

A higher reduction on the viability of B16F10 tumour melanocytes in the presence of CM from RAW 264.7 treated with non-encapsulated and nano-encapsulated XN, relative to the absence of treatments, was observed, as shown in Figure 6, implying that the macrophage phenotype modulation is classic anti-tumour M1. PLGA nanoparticles stimulated a M1 macrophage phenotype, as well. (Figure 6). The results suggest that RAW 264.7 macrophages are a biological target for XN in the non-encapsulated and in the encapsulated form of PLGA-loaded nanoparticles, exhibiting a predominant M1 or antitumour phenotype.

It should be noted that the results obtained should take into account the effect of solubilized XN on B16F10 in the CM from RAW 264.7 that was previously treated with XN solution and XN-loaded PLGA nanoparticle suspension, which was found to be toxic to these tumour cells. Therefore, the observed decrease in B16F10 viability may result from the association between the direct cytotoxic effect of solubilized XN and the anti-tumour effect promoted by RAW 264.7 stimulated with the mentioned treatments. This additive or synergistic effect simulates what happens in vivo, as XN will act simultaneously on the tumour cells of cutaneous melanoma and on the microenvironment components, namely macrophages.

### 3.8. RAW 264.7 Phenotype Membrane Markers Study with Previous Stimulation with XN Solubilized Form and Non-Encapsulated PLGA Nanoparticles

**Figure 7:**
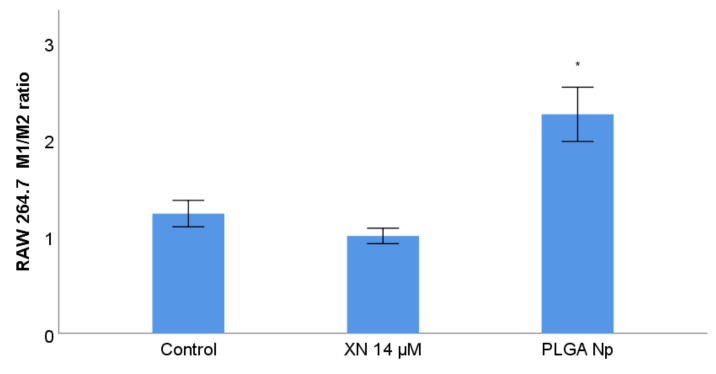
RAW 264.7 phenotype membrane markers study with previous stimulation with XN solubilized form and PLGA nanoparticles. The control refers to RAW 264.7 incubated with DMEM. “XN”, “Np” and “PLGA” stands for XN, nanoparticles and poly-lactic-co-glycolic acid, respectively. M1 represents an anti-tumour phenotype and M2 a pro-tumour phenotype. M1/M2 ratio values are expressed as mean ± S.E.M.(*n* = 6 for each treatment assay).* Statistically different at a level of *p* < 0.050 in comparison with the control.

To examine whether free and encapsulated XN induced a macrophage phenotypic switch, immunofluorescence was performed to quantify the CD86 (M1 polarization) and CD206 (M2 polarization) macrophage membrane markers of RAW 264.7 treated with non-encapsulated XN and PLGA nanoparticles. XN and PLGA nanoparticles have a macrophage phenotype modulation capacity per se (Figure 7). They induce M2 and M1 polarization phenotypes on RAW 264.7, respectively (Figure 7), with statistically significant differences between both phenotypes. This XN-induced M2 polarization is compatible with the anti-inflammatory activity of this polyphenol [68,69,70]. The M1-type polarization observed for free PLGA nanoparticles may be directly induced by PLGA and/or PVA, although only a few studies support this hypothesis [71]. The highest reduction in B16F10 melanoma cell viability observed upon incubation with conditioned medium from PLGA nanoparticles-treated RAW 264.7 can be explained by the previous findings that PLGA nanoparticles induce a switch towards anti-tumor M1 phenotype. The antitumor phenotype of these cells may thereby reduce B16F10 melanoma cell viability.

Comparing the results of this assay with those of the previous one, it is suggested, on the one hand, that XN has a more significant cytotoxic action than its macrophage immunomodulatory effect at the tested concentration of 14 µM, and, on the other hand, that PLGA polymeric nanoparticles have a more significant macrophage immunomodulation activity than their observed cytostimulant effect, for a tested concentration of nanoparticles equivalent to 14 µM. The total effect of XN-loaded PLGA nanoparticles on B16F10, in the presence of macrophages stimulated by this nanosystem, is the result of the association between the action on cell viability and the immunomodulatory action, both exerted by the individual components of the nanosystem. It was essential to design the in vitro test performed to understand all the biological activities tested in order to optimize the formulation of the produced nanosystem. The optimization will minimize the pro-tumour effect of macrophages of the tumour microenvironment, so that the action of nanoparticles loaded with XN can be a therapeutic adjuvant against malignant cutaneous melanoma. Probably the same components that induce a macrophage M1 phenotype are those that cause increased viability of tumour melanocytes when PLGA nanoparticles are tested. To minimize this increase in melanoma viability, and the selection of one that does not cause tumour growth and maintains or enhances M1 or antitumour macrophage modulation. Further testing should be done to test new nanoparticle formulations and new encapsulation methods for XN to optimize the interaction between these nanosystems, B16F10 tumour melanocytes and RAW 264.7 macrophages in novel in vitro models. To maximize the cytotoxic effect of the nanoparticles used in this study, their surface may be functionalized to make them selective for the melanoma site.

## 4. Conclusions

The XN was successfully encapsulated in PLGA nanoparticles, and the formulation displayed an antitumour effect by presenting cytoxicity and inhibiting the proliferation and migration of the B16F10 malignant cutaneous melanoma cell line. These results indicate that XN delivered by PLGA nanoparticles is an alternative with high therapeutic and preventive potential against malignant cutaneous melanoma. However, the underlying mechanisms of XN and XN-loaded PLGA nanoparticles are still unknown. A pertinent and interesting future approach will be to perform the same tests in in vitro and in vivo models simulating pathological metabolic conditions such as obesity. Precision medicine has gained relevance in the scientific community and extending studies on nanotechnology to metabolic risk groups is very valuable to design personalized antitumour therapies.

## Figures and Tables

**Table 1:** Diameter (nm), PdI, and ZP (mV), AE, and LC of the XN-loaded PLGA nanoparticles and unloaded nanoparticles (*n* = 3, mean ± SD).

Formulation	Size (nm)	PdI	ZP (mV)	AE (%)	LC (%)
PLGA Np	273 ± 18	0.285 ± 0.015	−15.4 ± 2.1	-	-
XN-PLGA Np	312 ± 49	0.259 ± 0.015	−18.2 ± 1.4	88.7 ± 4.3	15.9 ± 1.1

## Data Availability

Not applicable.
